# Perfil Clínico, Laboratorial e de Métodos de Imagem na Amiloidose Sistêmica em um Centro de Referência Cardiológico Brasileiro

**DOI:** 10.36660/abc.20201003

**Published:** 2021-12-20

**Authors:** Fábio Fernandes, Aristóteles Comte de Alencar, Bruno Vaz Kerges Bueno, Caio Rebouças Fonseca Cafezeiro, João Henrique Rissato, Roberta Shcolnik Szor, Mariana Lombardi Peres de Carvalho, Wilson Mathias, Angelina Maria Martins Lino, Jussara Bianchi Castelli, Evandro de Oliveira Souza, Félix José Alvarez Ramires, Viviane Tiemi Hotta, José Soares, Caio de Assis Moura Tavares, José Eduardo Krieger, Carlos Eduardo Rochitte, André Dabarian, Ludhmila Abrahão Hajjar, Roberto Kalil, Charles Mady

**Affiliations:** 1 Hospital das Clínicas Faculdade de Medicina Universidade de São Paulo São Paulo SP Brasil Instituto do Coração do Hospital das Clínicas da Faculdade de Medicina da Universidade de São Paulo, São Paulo, SP – Brasil; 2 Hospital das Clínicas Faculdade de Medicina Universidade de São Paulo São Paulo SP Brasil Hospital das Clínicas da Faculdade de Medicina da Universidade de São Paulo, São Paulo, SP – Brasil; 3 Universidade de São Paulo Instituto do Câncer de São Paulo Octavio Frias de Oliveira São Paulo SP Brasil Universidade de São Paulo - Instituto do Câncer de São Paulo Octavio Frias de Oliveira, São Paulo, SP – Brasil

**Keywords:** Amiloidose, Cadeias Leves de Imunoglobulina, Insuficiência Cardíaca, Cardiomiopatia Hipertrófica, Diagnóstico por Imagem

## Abstract

**Fundamento:**

Amiloidose sistêmica é uma doença com manifestações clínicas diversas. O diagnóstico envolve suspeita clínica, aliada a métodos complementares.

**Objetivo:**

Descrever o perfil clínico, laboratorial, eletrocardiográfico e de imagem no acometimento cardíaco da amiloidose sistêmica.

**Métodos:**

Estudo de uma amostra de conveniência, analisando dados clínicos, laboratoriais, eletrocardiográficos, ecocardiográficos, medicina nuclear e ressonância magnética. Considerou-se significância estatística quando p < 0,05.

**Resultados:**

Avaliaram-se 105 pacientes (com mediana de idade de 66 anos), sendo 62 homens, dos quais 83 indivíduos apresentavam amiloidose por transtirretina (ATTR) e 22 amiloidose por cadeia leve (AL). Na ATTR, 68,7% eram de caráter hereditário (ATTRh) e 31,3% do tipo selvagem (ATTRw). As mutações mais prevalentes foram Val142Ile (45,6%) e Val50Met (40,3%). O tempo de início dos sintomas ao diagnóstico foi 0,54 e 2,15 anos nas formas AL e ATTR (p < 0,001), respectivamente. O acometimento cardíaco foi observado em 77,9% dos ATTR e 90,9% dos AL. Observaram-se alterações de condução atrioventricular em 20% e intraventricular em 27,6% dos pacientes, sendo 33,7 % na ATTR e 4,5% das AL (p = 0,006). A forma ATTRw apresentou mais arritmias atriais que os ATTRh (61,5% x 22,8%; p = 0,001). Ao ecocardiograma a mediana da espessura do septo na ATTRw x ATTRh x AL foi de 15 mm x 12 mm x 11 mm (p = 0,193). Observou-se BNP elevado em 89,5% dos indivíduos (mediana 249 ng/mL, IQR 597,7) e elevação da troponina em 43,2%.

**Conclusão:**

Foi possível caracterizar, em nosso meio, o acometimento cardíaco na amiloidose sistêmica, em seus diferentes subtipos, através da história clínica e dos métodos diagnósticos descritos.

## Introdução

A amiloidose refere-se a um conjunto de doenças raras, no qual fragmentos de proteínas, dobrados em configuração altamente estável (folhas com pregueado do tipo “beta”), depositam-se de forma patogênica no espaço extracelular de órgãos e tecidos como fibrilas insolúveis. Conforme o tipo de polímeros de subunidades (monômeros) e proteína depositada, temos diferentes subtipos de doença.

O acometimento cardíaco é frequente, manifestando-se com espessamento e desarranjo estrutural, podendo ocasionar disfunção diastólica e sistólica, síndrome de insuficiência cardíaca (IC), distúrbios de condução e arritmias atriais e ventriculares com elevada morbimortalidade. Entre todas as formas de amiloidose conhecidas (36 até o momento), a maioria dos casos de amiloidose cardíaca deve-se ao depósito duas proteínas: cadeias leves (AL) ou transtirretina (ATTR).^1-3^

A forma sistêmica mais comum é causada por depósitos de cadeias leves, que se referem aos clones dessas cadeias e associados a anticorpos formados por clones de plasmócitos na medula óssea (amiloidose AL). Por sua vez, a transtirretina é uma proteína produzida no fígado, sendo carreadora de tiroxina e retinol. Sua forma monomérica é mais propensa a dobrar erroneamente e depositar-se nos tecidos, gerando amiloidose.

Há dois subtipos principais de amiloidose por transtirretina (ATTR): a ATTR selvagem (ATTR *wild type*, ou ATTRw), definida até recentemente como “senil”, na qual fragmentos de uma proteína produzida normalmente acumulam-se nos tecidos ao longo de anos; e a ATTR mutante (ATTR hereditária ou ATTRh), na qual indivíduos são portadores de mutações patológicas no gene da transtirretina, que predispõe ao depósito acelerado dessas proteínas. Existem mais de 120 mutações descritas até o momento.^1,3^

No entanto, estabelecer o diagnóstico de amiloidose cardíaca é difícil e requer alto índice de suspeita clínica. Nesse contexto, os exames de imagem, principalmente o ecocardiograma, a cintilografia e a ressonância magnética cardíaca, têm contribuído cada vez mais para o reconhecimento do depósito amiloide.

O diagnóstico correto se faz necessário, pois o tratamento habitual para insuficiência cardíaca nem sempre pode ser aplicado na cardiopatia amiloidótica. O prognóstico é diferente das demais etiologias; a evolução e o tratamento são diferentes das demais cardiomiopatias hipertróficas, principalmente pelas possibilidades terapêuticas específicas atuais, que podem modificar a história natural da doença. Além disso, convém ressaltar que o diagnóstico de amiloidose AL é uma emergência cardiológica.

De modo geral, estudos demonstram que o diagnóstico da doença sistêmica é tardio; o tempo entre o início dos sintomas até o estabelecimento do diagnóstico é de dois anos na forma AL e de até quatro anos na ATTR. Cerca de 32% dos pacientes foram atendidos por até cinco diferentes médicos para chegar ao diagnóstico, com 39% apresentando neuropatia limitante, o que comprometia o tratamento da doença.^4,5^ No entanto, não existem dados específicos sobre o tempo de início dos sintomas cardiovasculares e o diagnóstico da doença na população brasileira.

## Objetivo

Descrever o perfil clínico, laboratorial, eletrocardiográfico e de métodos de imagem em um grupo de pacientes com amiloidose sistêmica encaminhados a um centro de referência cardiológico, para uma melhor compreensão das características da doença no Brasil, propiciando a criação de novas estratégias de manejo.

## Métodos

### Desenho e população de estudo

**Figura 1:**
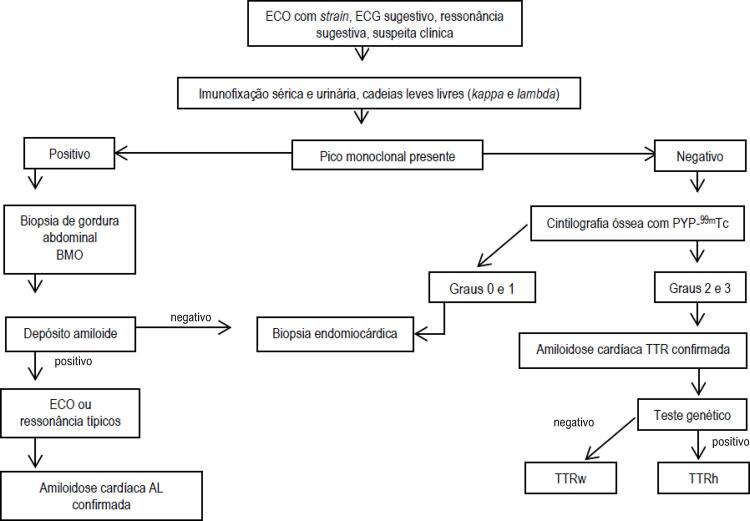
– Fluxograma de diagnóstico da amiloidose cardíaca (adaptado de referência 8). BMO: biopsia de medula óssea; TTRw: amiloidose por transtirretina, forma selvagem; TTRh: amiloidose por transtirretina, forma hereditária; ECG: etrocardiograma.

O estudo incluiu pacientes acompanhados em nosso serviço, com diagnóstico confirmado de amiloidose sistêmica com miocardiopatia, e indivíduos oriundos de outras unidades de saúde e diferentes especialidades (neurologia, hematologia, nefrologia e gastrenterologia) para avaliação de acometimento cardíaco (cardiomiopatia amiloidótica) de doença já confirmada em outros órgãos e sistemas. No seguimento ambulatorial, os pacientes foram submetidos à pesquisa de amiloidose, conforme fluxograma internacional atualmente adotado (Figura 1),^6^ através de eletrocardiograma, ecocardiograma, cintilografia óssea com PYP-^99m^Tc, ressonância magnética cardíaca, eletroforese de proteínas com imunofixação, dosagem de cadeias leves livres séricas e pesquisa genética de mutações da transtirretina. Também foi realizado o rastreamento por genotipagem dos familiares dos pacientes com amiloidose hereditária. Os dados foram coletados no período de janeiro de 2018 até maio de 2020.

### Critérios de inclusão e exclusão

Incluíram-se na presente amostra pacientes com fenótipo clínico, laboratorial e de imagem com suspeita de amiloidose. Os indivíduos foram encaminhados para avaliação ou acompanhados em nosso serviço.

Excluíram-se do estudo pacientes sem comprovação de amiloidose pelos métodos descritos, pacientes que aguardavam exames diagnósticos comprobatórios e indivíduos com outras formas de cardiopatias, como hipertensivas, isquêmicas, chagásicas, além de portadores de doença valvar (estenose aórtica) concomitante.

### Critérios diagnósticos

Os casos de amiloidose por transtirretina foram definidos pela ausência de componente monoclonal, identificado por eletroforese com imunofixação de proteínas séricas e urinárias, dosagem e relação normal das cadeias leves livres séricas (*kappa* e *lambda*) e presença de captação miocárdica graus II ou III de Perugini na cintilografia miocárdica com pirofosfato marcado com tecnécio 99 metaestável (PYP-^99m^Tc).^7^ Nos casos de forma AL, o diagnóstico de amiloidose foi confirmado histologicamente através de biopsia de gordura abdominal, medula óssea, gengiva ou miocárdio. Inicialmente, houve comprovação do componente monoclonal por um dos métodos descritos acima, além de captação miocárdica ausente ou grau I na cintilografia.

### Métodos de imagem

Os estudos ecocardiográficos foram realizados com o uso do equipamento Vivid^TM^E95, da GE Healthcare (Waukesha, Wisconsin). As avaliações foram feitas em modo bidimensional e tridimensional em tempo real, com a análise de fluxos cardíacos por dopplerecocardiografia, análise com *doppler* tecidual, análise por técnica de *speckle tracking* da deformação miocárdica (*strain*) longitudinal global bidimensional do ventrículo esquerdo e análise da deformação miocárdica (*strain*) do ventrículo direito. As imagens foram adquiridas em projeções paraesternais longitudinais de câmaras esquerdas, transversal e apical de 2, 3 e 4 câmaras, de acordo com padronização da Sociedade Americana de Ecocardiografia. As aquisições das imagens tridimensionais em tempo real foram obtidas em apneia expiratória, com observação do ciclo cardíaco a partir do registro eletrocardiográfico.

As cintilografias miocárdicas com PYP-^99m^Tc foram realizadas através da captação de imagens após 1 e 3 horas da injeção do traçador, mediante o uso de um colimador de baixa energia e alta resolução (LEHR). Utilizaram-se projeção estática anterior (1 hora e 3 horas, respectivamente) e projeções estáticas anterior, lateral esquerda e oblíqua anterior esquerda (3 horas). Quando houve captação na área cardíaca, realizaram-se imagens SPECT de tórax, com tempo de 3 horas de injeção, para melhor identificar os limites cardíacos e evitar que o *pool* interferisse na interpretação das imagens. Isso acarretaria resultados falso-positivos. Utilizaram-se dois critérios de interpretação das imagens: visual e análise semiquantitativa. O critério visual baseou-se na escala de Perugini, na qual foi feita uma análise qualitativa nas imagens de 3 horas, comparando o grau de concentração cardíaca do traçador com o de captação óssea do gradil costal. No grau 2, a concentração é semelhante em ambos os pontos avaliados; no grau 3, a captação cardíaca é maior do que a observada nos arcos costais. Ambas estão fortemente associadas ao diagnóstico de amiloidose cardíaca por transtirretina. Na ausência de captação (grau 0), considerou-se que não havia depósito amiloide cardíaco; na captação cardíaca discreta, menor que nas costelas (grau 1), considerou-se a possibilidade de depósito amiloide por cadeias leves (AL) ou fase inicial de depósito por transtirretina. Foram realizadas análises semiquantitativas do grau de captação do radiotraçador, comparando-se as regiões de interesse (ROIs) da área cardíaca e da área em espelho no hemitórax direito na imagem anterior. Considerou-se seriamente o sugestivo de amiloidose por transtirretina e valores de relação coração/lado contralateral ≥ 1,5 nas imagens de 1 hora e ≥ 1,3 nas imagens de 3 horas. Os critérios utilizados estão em conformidade com o protocolo da American Society of Nuclear Cardiology e a diretriz de cintilografia com marcadores ósseos da Sociedade Brasileira de Medicina Nuclear.^8,9^

As ressonâncias magnéticas cardíacas foram realizadas em aparelhos de 1,5 Tesla (Achieva, Philips Medical Systems, Best, The Netherlands Signa CV/I; GE Medical Systems Wakeusha, WI; e Avanto; Siemens Medical Solutions, Erlangen, Germany), com as imagens sendo captadas nos eixos curto e longo em apneia e em sequências de pulso sincronizadas com o eletrocardiograma. A primeira sequência foi um gradiente-eco em estado de equilíbrio (*steady-state free precession* – SSFP) para avaliar a morfologia e as funções ventriculares esquerda e direita. A segunda sequência foi um gradiente-eco segmentado com pulso de inversão-recuperação para obter o RTM, 10 a 20 minutos após a administração intravenosa de 0,2 mmol/kg de bolo de contraste à base de gadolínio (Dotarem^®^, ácido gadotérico – Gd-DOTA, Guerbet Aulnay-Sous-Bois – França). Para as imagens de cine com uso da sequência SSFP, os parâmetros foram: tempo de repetição, 3,4 ms; tempo de eco, 2,0 ms; ângulo de deflexão, 45º; matriz, 256x160; fases cardíacas,^20^ cortes por segmento, 8 a 16 para obter resolução temporal de 55 ms ou menos; espessura do corte, 8 mm; intervalo entre os cortes, 2 mm; e campo de visão, 36 a 40 cm. Para a sequência de pulso do RTM, os seguintes parâmetros foram usados nos eixos curto e longo: tempo de repetição, 7,3 ms; tempo de eco, 3,2 ms; ângulo de deflexão, 25º; matriz, 256 x 196; espessura do corte, 8 mm; intervalo entre os cortes, 2 mm; campo de visão, 36 a 40 cm; tempo de inversão, 200 ms a 300 ms; largura da banda receptora, 32,5 kHz; cada aquisição RR; e número de excitações, 2. Os cortes no eixo curto foram prescritos da base ao ápice (em geral 8 a 12 cinecortes/coração) e perpendicular ao eixo longo ventricular, cobrindo todo o ventrículo esquerdo. Importante notar que as localizações dos cortes foram exatamente as mesmas para as duas sequências de pulso, permitindo comparar função e morfologia, com a caracterização tecidual fornecida por RTM.

### Variáveis analisadas

No presente estudo, foram avaliados os critérios idade, sexo, tempo entre o início de sintomas até a confirmação do diagnóstico, sistema inicialmente acometido (cardiológico, neurológico, ambos ou outros), alterações no eletrocardiograma e/ou Holter de 24 horas (ritmo cardíaco, baixa voltagem, sobrecargas de câmaras, distúrbios de condução atrioventricular ou intraventricular e arritmias evidenciadas), dados do ecocardiograma transtorácico (espessura de septo interventricular, fração de ejeção do ventrículo esquerdo pelo método de Simpson, padrão de diástole, alteração (ou não) valvar e discinesias poupando o ápice do ventrículo (padrão de *apical sparing*)), dados de ressonância magnética cardíaca, dados de cintilografia através de pirofosfato marcado com ^99m^Tc (grau de captação do marcador e razão de captação da região cardíaca com a região torácica direita após uma e três horas), valores de BNP ou NT-pro-BNP, detecção de elevação ou não de troponina sérica, presença ou não de componentes monoclonais em imunofixação de proteínas séricas e urinárias, dosagens de cadeias leves livres séricas e a relação entre as mesmas.

Foram considerados os seguintes sintomas cardiovasculares: palpitações, dor torácica, hipotensão sintomática, hipotensão ortostática, síncope, dispneia aos esforços e ortopneia associada a edema de membros inferiores. A insuficiência cardíaca foi definida quando constatadas a turgência jugular patológica, o refluxo hepatojugular, a dispneia paroxística noturna, a estertoração pulmonar, a presença de terceira bulha, o edema de membros inferiores bilateral e dispneia aos esforços.

### Análise estatística

O tamanho da amostra empregada no estudo foi definido por conveniência. As variáveis contínuas com distribuição normal foram descritas através de média e desvio-padrão, as variáveis contínuas sem distribuição normal foram descritas através de mediana e intervalo interquartil.

Para comparar as variáveis quantitativas idade, tempo de doença clínica, espessura do septo no ecocardiograma, fração de ejeção do ventrículo esquerdo, valores de captação na cintilografia com PYP-^99m^Tc e níveis séricos de BNP e NT-pro-BNP, utilizou-se o teste de Mann-Whitney. Asseveramos a normalidade das variáveis quantitativas de desfecho principal através do teste de Kolmogorov-Smirnov (KS) e concluímos que não existe distribuição de normalidade assegurada.

Para a análise da dependência estatística e da distribuição de frequências das variáveis qualitativas sexo, alterações no ECG e Holter de 24 horas, presença (ou não) de picos monoclonais nas imunofixações, alterações valvares na espessura de septo e na diástole ao ecocardiograma, presença (ou não) de realce tardio na ressonância e elevação (ou não) de troponina, aplicou-se o teste de Qui-quadrado.

Para comparar a proporção de respostas entre as duas variáveis, empregou-se o teste de Igualdade de Duas Proporções. A comparação entre os grupos AL, ATTRh e ATTRw para as variáveis quantitativas foi realizada através do teste de Kruskal-Wallis.

Os *softwares* estatísticos utilizados para as análises foram o SPSS versão 20 (IBM Corp., Armonk, NY) e o Minitab 16 (Minitab, LLC). Todos os testes foram considerados significantes estatisticamente quando p < 0,05.

O projeto foi aprovado pela Comissão Científica e de Ética do Instituto do Coração do Hospital das Clínicas, da Faculdade de Medicina da Universidade de São Paulo (InCor/HC-FMUSP); e pela Comissão de Ética para a Análise de Projetos de Pesquisa (CAPPesq), da Diretoria Clínica do Hospital das Clínicas da Universidade de São Paulo (CAAE n^o^ 27437019.5.0000.0068).

## Resultados

### Perfil epidemiológico

Foram avaliados 105 pacientes, com mediana de idade de 66 anos, sendo 64 anos na forma ATTR e 66 anos na forma AL. Avaliando-se os subtipos da amiloidose por transtirretina, a mediana de idade na forma ATTRh foi de 56 anos, enquanto na ATTRw foi de 79 anos.

Em nosso estudo, 62 pacientes (59%) eram do sexo masculino, sendo que 54 indivíduos apresentavam forma ATTR (34 ATTRh e 20 ATTRw) e 8 apresentavam forma AL. Entre as 43 pacientes do sexo feminino, 29 tinham forma ATTR (23 ATTRh e 6 ATTRw) e 14 tinham forma AL.

### Tipos de amiloidose

**Figura 2:**
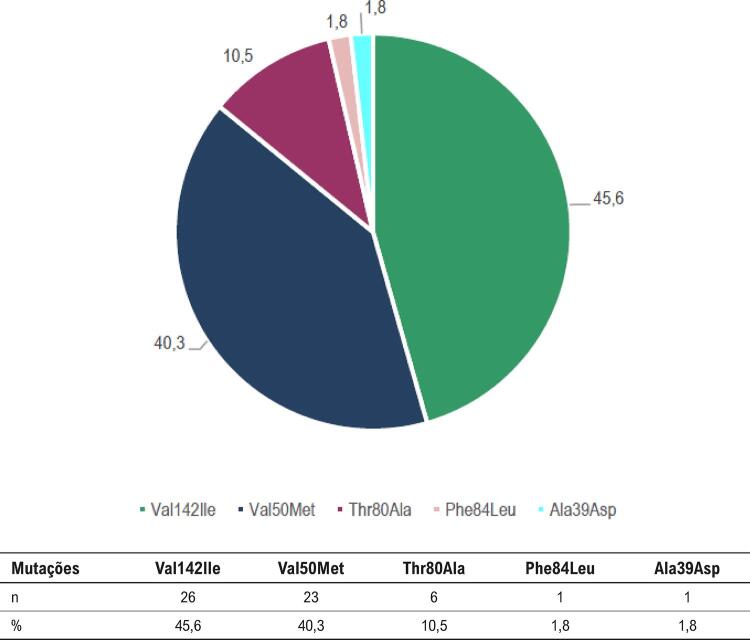
– Distribuições das mutações encontradas na ATTRh. Val50Met: substituição da valina pela metionina na posição 50; Thr80Ala: substituição da treonina pela alanina na posição 80; Ala39Asp: troca alanina pelo aspartato na posição 39; Phe84Leu: substituição da fenilalanina pela leucina na posição 84.

O subtipo de amiloidose mais prevalente foi a secundária à mutação da transtirretina (ATTR), sendo que a forma hereditária (ATTRh) foi a mais comum. Observaram-se cinco mutações diferentes, sendo a Val142Ile (substituição do aminoácido valina na posição 142 pela isoleucina) a mais frequente, seguida pela Val50Met (substituição do aminoácido valina na posição 50 pela metionina), como observado na Figura 2.

### Tempo até o diagnóstico

O tempo entre início dos sintomas até o diagnóstico foi, em média, de 0,54 ± 1,94 anos na forma AL e de 2,15 ± 2,43 anos na ATTR (p < 0,001). No subtipo ATTRh, o tempo médio foi de 16 meses e na ATTRw foi de 37 meses (p < 0,049).

### Apresentação clínica

Na primeira consulta, a apresentação clínica constituiu-se principalmente de sintomas cardiológicos, isoladamente ou associados a sintomas neurológicos (polineuropatia periférica, alterações de hábito gastrintestinal, síndrome do túnel do carpo, alterações vesicais), independente da forma de amiloidose (Tabela 1).

**Tabela 1:** – Apresentação clínica nos diferentes subtipos de amiloidose

Sistemas acometidos	AL		ATTR		Total	
	N	%	N	%	N	%
Assintomático	3	15,80%	14	18,70%	17	18,10%
Cardiovascular	15	78,90%	41	54,70%	56	59,60%
Neurológico	1	5,30%	15	20,00%	16	17,00%
Misto	0	0,00%	5	6,70%	5	5,30%

*Total de pacientes: 105; sem dados de sintomas iniciais: 11; P: 0,189; AL: amiloidose de cadeias leves; ATTR: amiloidose por transtirretina.*

Observou-se que 31% dos nossos pacientes apresentavam associação de neuropatia dolorosa, distúrbios de marcha, síndrome do túnel do carpo bilateral ou disautonomia a hipertrofia ventricular e/ou ICFEp.

Em nosso estudo, 17 pacientes eram assintomáticos, o que ocorreu devido ao rastreio de familiares de probandos com ATTRh. O sintoma mais prevalente foi a dispneia isolada (38%) ou associada a sintomas como incontinência urinária, polineuropatia, tontura, disautonomia e síncope (16%). Em 5% dos casos, o primeiro sintoma foram palpitações, em geral relacionadas a diagnóstico posterior de arritmia cardíaca.

### Biomarcadores

Observou-se elevação dos níveis séricos de BNP em 94 pacientes (89,5%, mediana 249 ng/mL, IQR 597,7) sendo maior nos pacientes com ATTR que nos indivíduos com a forma AL. Os níveis séricos de troponina apurados estavam acima do limite superior da normalidade em 43,2% dos pacientes estudados.

A eletroforese de proteínas séricas ou urinárias evidenciou picos monoclonais em somente três pacientes com ATTR, sendo considerada uma gamopatia de valor incerto, após serem submetidos à avaliação hematológica e à biopsia de medula óssea.

### Eletrocardiograma de 12 derivações (ECG) e Holter de 24 horas

Mais da metade dos pacientes apresentaram algum tipo de alteração eletrocardiográfica. Observou-se que 1/5 dos pacientes apresentavam ritmo diferente do sinusal ao ECG, sendo a fibrilação atrial (FA) o mais encontrado. Os demais ritmos observados foram o *flutter* atrial, o ritmo ectópico atrial e o ritmo de marcapasso (Tabelas 2 e 3).

**Tabela 2:** – Alterações eletrocardiográficas nos diferentes tipos de amiloidose

Achados	ECG alterado	BAV	BIV	Arritmia atrial	Arritmia ventricular	Fibrilação atrial
Total	72 (68,6%)	21(20%)	29 (27,6%)	36 (34,3%)	11 (10,5%)	16 (15,2%)
AL	14 (66,7%)	2 (9,1%)	1 (4,5%)	7 (31,8%)	3 (13,6%)	2 (9,1%)
ATTR	58 (80,6%)	19(22,9%)	28(33,7%)	29 (34,9%)	8 (9,6%)	14 (16,9%)
p	0,180	0,150	0,006	0,784	0,586	0,719

*AL: amiloidose de cadeias leves; ATTR: amiloidose por transtirretina; ECG: eletrocardiograma; BAV: bloqueio atrioventricular; BIV: bloqueio intraventricular. Método: teste de Qui-quadrado.*

**Tabela 3:** – Alterações eletrocardiográficas nos subtipos ATTR

Achados	ECG alterado n (%)	BAVs n (%)	BIVs n (%)	Arritmia atrial n (%)	Arritmia ventricular n (%)	Fibrilação atrial n (%)
ATTR	58 (80,6)	19 (22,9)	28 (33,7)	29 (34,9)	8 (9,6)	14 (16,9)
ATTRh	36 (76,6)	11 (19,3)	17 (29,8)	13 (22,8)	4 (7)	4 (7)
ATTRw	22 (88)	8 (30,8)	11 (42,3)	16 (61,5)	4 (15,4)	10 (38,5)
p	0,244	0,249	0,265	0,001	0,231	0,003

*ECG: eletrocardiograma; BAV: bloqueio atrioventricular; BIV: bloqueio intraventricular; ATTR: amiloidose por transtirretina; ATTRh: amiloidose por transtirretina, forma hereditária; ATTRw: amiloidose por transtirretina, forma selvagem. Método: teste de Qui-quadrado.*

Além das arritmias sustentadas persistentes, também foi constatada, em Holter, a presença de extrassistolia atrial e taquicardia atrial paroxística em 12 pacientes, e extrassistolia ventricular, ritmo idioventricular acelerado e taquicardia ventricular não sustentada em 14 indivíduos, com sobreposição de arritmia em alguns deles.

Foram encontrados distúrbios de condução em quase metade dos pacientes. A alteração de condução atrioventricular mais comumente encontrada foi o bloqueio de primeiro grau, enquanto 5 pacientes necessitaram de implante de marcapasso cardíaco. Avaliando a condução intraventricular (bloqueios de ramos e bloqueios divisionais), observou-se que a presença de distúrbio de condução foi muito mais frequente na forma de amiloidose ATTR quando comparada a forma AL (Tabelas 2 e 3). Em 20 pacientes (19,1%), observou-se baixa voltagem (amplitude de QRS < 5 mm nas derivações clássicas ou < 10 mm nas precordiais); e 21 indivíduos (20%) possuíam padrão de pseudoinfarto ao ECG (definido como presença de ondas Q patológicas em pelo menos duas derivações contíguas no eletrocardiograma, sem doença coronariana obstrutiva).

### Ecocardiograma

A fração de ejeção do ventrículo esquerdo (FEVE) foi maior em valores absolutos nos pacientes com a forma AL do que na forma TTR, porém sem diferença estatística significativa entre os tipos (Tabela 4).

**Tabela 4:** – Parâmetros ecocardiográficos nos subtipos amiloidose

Parâmetros	ATTRh Mediana (IQR)	ATTRw Mediana (IQR)	AL Mediana (IQR)	p
Septo (mm)	12 (7,3)	15 (6,5)	11 (4,0)	0,193
Parede posterior (mm)	11 (5,8)	12 (4,5)	11 (2,0)	0,531
Diâmetro diastólico (mm)	45 (5,5)	49,5 (13)	44 (8,0)	**0,012**
Diâmetro sistólico (mm)	30 (6,0)	32 (14)	29 (7,0)	0,055
FEVE (%)	60,5 (26)	58,5 (19,3)	62 (16)	0,230
Diâmetro AE (mm)	42 (9,0)	45 (7,8)	38,5 (9,3)	**0,001**
PSAP (mmHg)	32 (11,5)	34 (15)	33 (10,8)	0,813

*IQR: intervalo interquartil; FEVE: fração de ejeção do ventrículo esquerdo; PSAP: pressão sistólica de artéria pulmonar; ATTRh: amiloidose por transtirretina forma hereditária; ATTRw: amiloidose por transtirretina forma selvagem; AL: amiloidose de cadeias leves. Análises pelo teste de Mann-Whitney.*

Metade dos pacientes apresentavam espessamento do septo interventricular, sendo que o padrão de hipertrofia assimétrica e simétrica foram igualmente encontrados. Quando analisado por subtipo de amiloidose, observaram-se mais casos de hipertrofia assimétrica na forma ATTR quando comparada a AL, sem significância estatística.

Tanto as medidas do diâmetro diastólico do ventrículo esquerdo quanto as de diâmetro do átrio esquerdo foram maiores nos pacientes com ATTRw, quando comparadas a ATTRh e AL, apresentando significância estatística (Tabela 4). Não houve diferença quanto a espessura do septo interventricular, diâmetro sistólico do ventrículo esquerdo ou pressão sistólica da artéria pulmonar (PSAP).

Nos pacientes analisados com o método de *strain* (n = 22), 81,8% apresentaram padrão de preservação apical *(apical sparing*). Cerca de 53% dos casos manifestaram sintomas de insuficiência cardíaca com fração de ejeção do ventrículo esquerda preservada. Dos 16 pacientes que apresentavam sintomas exclusivamente neurológicos no início da avaliação, cinco apresentavam hipertrofia ventricular ao ecocardiograma.

### Ressonância magnética

Dos 105 pacientes do estudo, 58 foram submetidos a avaliação por ressonância magnética, dos quais 24 (41%) apresentavam hipertrofia ventricular esquerda, 17 com a forma ATTR e 7 com a forma AL. Na forma ATTR, 10 eram ATTRh e 7 ATTRw. A presença de realce tardio de padrão não isquêmico foi observada em 38 pacientes (68%), independentemente da existência de hipertrofia ventricular. A quantificação do volume extracelular e do mapa T1 não foi realizada de rotina, não sendo possível elaborar uma análise.

### Medicina nuclear

A cintilografia com PYP-^99m^Tc foi realizada em 66 pacientes. Em 40 indivíduos (60%), ela foi considerada positiva, pelos critérios previamente descritos. Observou-se que, na análise após 3 horas, o grau de captação foi significativamente maior nos pacientes com ATTR (mediana 1,54; IQR 0,42) do que nos com forma AL (mediana 1,18; IQR 0,02) (p = 0,028). Na ATTRw, a mediana da captação foi de 1,6 (IQR 0,29), maior do que na ATTRh, com mediana de 1,27 (IQR 0,6), porém não sendo estatisticamente significante (p = 0,044). Em 12 pacientes com forma ATTRh, não houve nenhuma ou somente discreta captação, e em 3 indivíduos o exame foi considerado indeterminado, por haver padrão discordante entre a análise visual e a semiquantitativa.

Levando-se em consideração todas as variáveis cardíacas avaliadas, apenas 18 indivíduos não apresentavam nenhuma alteração, sendo dois pacientes com forma AL e 16 pacientes com ATTRh. Nesse estrato, a maioria dos avaliados era familiar a casos considerados índices.

## Discussão

Em nossa casuística, analisamos dados adquiridos nos últimos 2 anos e caracterizamos 105 pacientes com amiloidose. Nesses indivíduos, o comprometimento cardíaco esteve presente em 83% dos casos, mesmo nas formas hereditárias que não acometem o coração como foco principal (Val50Met, por exemplo), corroborando para os dados que mostram este órgão como alvo da doença. Conforme levantamento dos dados da literatura, trata-se do estudo brasileiro com a maior casuística de pacientes cardiológicos até o momento.

A maior casuística nacional publicada, de Cruz et al., incluiu 160 pacientes. No entanto, apresentava predomínio de acometimento neurológico, com 35,2% de acometidos cardiológicos na amostra.^10^

Em nosso centro, a forma ATTRh foi a mais frequente, seguida pela selvagem (ATTRw) e AL.^11^ Nossa casuística difere da literatura mundial, em que a forma mais comum é a AL. O achado pode ser justificado pelo fato do nosso centro estar inserido em um hospital de referência cardiológica,^12^ enquanto os pacientes com a forma AL são referenciados para acompanhamento hematológico em outro instituto, dentro de um mesmo complexo hospitalar.

Entre todas as mutações descritas na ATTRh até o momento, a Val142Ile é a mais frequente nos Estados Unidos, encontrada em 3 a 4% dos indivíduos afro-americanos. Habitualmente, apresenta-se com sintomas de insuficiência cardíaca hipertrofia ventricular ao redor da sétima década de vida.^13^ No estudo, essa também foi a mutação mais prevalente, provavelmente por sermos um serviço de referência cardiológica, e demonstrou ser mais frequente do que a forma tardia da Val50Met.

Atualmente, entre os casos de ATTR, sabe-se que a forma selvagem é a mais prevalente no mundo, comumente subdiagnosticada.^12^ Acreditamos que, apesar dos avanços no diagnóstico da ATTR em nosso serviço, ainda há certa quantidade de casos ocultos, como os pacientes diagnosticados com estenose aórtica ou cardiomiopatia hipertrófica. Dois pacientes com mutação Val 142Ile estavam na coorte de pacientes com diagnóstico de cardiomiopatia hipertrófica (CMH), um deles com mutação homozigótica.

No Brasil e no mundo, a mutação Val50Met é a mais prevalente.^14,15^ Por vezes, com padrão etário bimodal em alguns países, apresenta fenótipo sobretudo neurológico quando acomete indivíduos mais jovens a partir dos 30 anos; e padrão misto neurológico e cardiológico no segundo pico de idade, a partir dos 50 anos.^14^ Por sermos um serviço terciário, o padrão observado nos pacientes com essa mutação, a segunda mais frequente em nosso estudo, foi o de acometimento misto a partir dos 50 anos.

A terceira mutação mais observada foi a Thr80Ala, descrita em pacientes ingleses e irlandeses com início dos sintomas ao redor dos 60 anos, fenótipo predominantemente cardíaco e de nervos autonômicos, com menor acometimento de neuropatia periférica e de pior prognóstico quando comparado à Val50Met.^16^

Em boa parte dos casos, a forma selvagem ocorre mais tardiamente que a hereditária. Em nosso estudo, a idade média da ATTRw foi 22 anos maior que hereditária, denotando pacientes mais idosos com mais comorbidades. Concluíram-se precocemente outras etiologias para sintomas cardiovasculares ou alterações estruturais ou eletrofisiológicas apresentadas, sem se cogitar a amiloidose como possibilidade etiológica.

O tempo entre o início dos sintomas e o diagnóstico foi mais prolongado na ATTR quando comparado à forma AL, possivelmente por esta última ter apresentação clínica mais exuberante, uma vez que as imunoglobulinas apresentam toxicidade direta ao tecido cardíaco.^5^ Comparando os subtipos, a ATTRw apresentou um tempo maior de doença até o diagnóstico que a ATTRh. Isso pode ser justificado pelo fato de a forma hereditária ocorrer em pacientes mais jovens, e eventualmente com índice na família já diagnosticado, gerando o rastreio familiar.

Nos pacientes com queixa clínica de insuficiência cardíaca, pouco mais da metade apresentou padrão de ICFEp, uma entidade frequente na prática clínica, especialmente nos indivíduos idosos, e que habitualmente é considerada apenas como disfunção diastólica relacionada à idade e a comorbidades associadas. Tal fator deve ser um dos sinais de alerta ao pensarmos na doença, principalmente quando associado a níveis elevados de biomarcadores.

Outros sinais e sintomas que devem levantar a suspeita diagnóstica são polineuropatia sensitivo-motora (neuropatia dolorosa, distúrbios de marcha e síndrome do túnel do carpo bilateral) ou disautonomia em pacientes com hipertrofia ventricular e/ou ICFEp, visto que 31% dos pacientes apresentavam tal associação, achado já descrito na literatura.^13^

Os biomarcadores BNP e troponina são importantes para demonstrar e quantificar a agressão miocárdica nos pacientes com amiloidose. Além disso, esses exames laboratoriais também são utilizados na avaliação prognóstica, tendo sido observados em parte dos nossos pacientes, denotando tanto a gravidade da doença quanto seu caráter de agressão contínua em atividade. Nossos dados corroboram dados da literatura, que identificam BNP, NT-proBNP e troponina como exames de potencial diagnóstico e prognóstico em casos suspeitos ou confirmados de amiloidose. A troponina alterada, sem quadro agudo isquêmico, inflamatório ou de outras doenças como fibrilação atrial e doença renal crônica, associado a sintomas de insuficiência cardíaca ou polineuropatia, deve ser um sinal de alerta para a amiloidose sistêmica.^10,12^

De modo geral, as alterações eletrocardiográficas são tardias, quando já existe um grande comprometimento cardíaco; no entanto, quando presentes, auxiliam no diagnóstico. O ECG sugere o diagnóstico quando se observam concomitantemente sinais de sobrecarga atrial esquerda, bloqueio atrioventricular de primeiro grau, padrão de pseudoárea inativa anterosseptal, perda de forças septais e baixa voltagem, especialmente quando esta é discordante da hipertrofia miocárdica encontrada no ecocardiograma ou ressonância cardíaca.^17^

O ECG e o Holter de 24 horas evidenciaram distúrbios de condução atrioventricular e intraventriculares em quase metade dos pacientes (Tabelas 2 e 3), seguidos de baixa voltagem e área inativa. Em nosso estudo, observamos maior incidência de alterações eletrocardiográficas do que em outro estudo brasileiro, no qual 56% dos pacientes apresentavam tal achado, provavelmente pelo maior número de pacientes com mutação de apresentação fenotípica predominantemente neurológica (Val50Met na forma precoce). Contudo, a incidência de distúrbios de condução assemelhou-se nos pacientes acometidos em ambos os trabalhos.^18^

Por tratar-se de doença de depósito infiltrativa e de caráter inflamatório, os distúrbios de condução são comumente encontrados. Dados da literatura sugerem que os bloqueios atrioventriculares estão entre as alterações mais frequentes, ocorrendo em 38% dos pacientes.^20^ Entretanto, em nosso estudo, não identificamos essa prevalência, possivelmente por termos incluído na análise formas mais precoces. Além disso, rastreamos ativamente portadores de mutação assintomáticos, familiares de pacientes com diagnóstico confirmado e mutações diferentes, incluídas nos diferentes estudos. Ao compararmos com outro estudo brasileiro, de Queiroz et al., feito com 51 pacientes, os dados foram mais semelhantes aos que encontramos: cerca de 13,7% dos indivíduos apresentavam bloqueios atrioventriculares e 19,6% bloqueios intraventriculares.^20^

A fibrilação atrial possui uma prevalência que cresce de forma exponencial com a idade, que os estudos epidemiológicos indicam ser de 3,7% a 4,2% nos indivíduos com idade entre 60 e 70 anos, e em torno de 7% a 8% em pacientes de 75 anos.^20,21^ Dessa forma, era esperado que a incidência de fibrilação nos pacientes com amiloidose cardíaca de forma selvagem em nossa corte fosse maior que na forma hereditária, pois havia diferença de idade no momento do diagnóstico da doença nos dois grupos. No entanto, ficou evidente a prevalência muito maior que os dados mundiais de população geral, pois 7% dos pacientes com forma ATTRh e 38,5% dos pacientes com a forma ATTRw apresentavam arritmia. Ainda assim, nossa prevalência foi menor do que a encontrada na corte de Donnellan et al.,^22^ que observou fibrilação atrial em 69% dos pacientes com amiloidose cardíaca por transtirretina. Tais achados sugerem que a fibrilação pode ser um marcador da doença, principalmente quando nos deparamos com pacientes sem causa aparente ou que justifiquem a arritmia.

Assim como na fibrilação atrial, as ectopias supraventriculares frequentes (definidas como > 30 ectopias por hora) e as taquicardias atriais são mais prevalentes com o aumento da idade, dado também evidenciado em nossa coorte, na qual 61,5% dos pacientes com a forma ATTRw apresentavam alguma dessas arritmias, isoladamente ou concomitantes.

Menos observadas do que as arritmias atriais nesta população, as arritmias ventriculares também são encontradas, com espectro que varia desde ectopias raras (definidas como < 10 ectopias por hora) até episódios de taquicardia ventricular, habitualmente não sustentada. Em um trabalho, foi observada em 17% dos pacientes arritmia ventricular complexa com amiloidose ATTR, e em 27% dos pacientes amiloidose AL. Em nosso estudo, também observamos maior incidência percentual de arritmia ventricular na forma AL (13,6%) frente à ATTR (9,6%), sem significância estatística e ambas com menor prevalência que o referido estudo.^23^ Dessa forma, a amiloidose também deve ser considerada como diagnóstico diferencial em pacientes com arritmias cardíacas sem causa aparente.

Os exames de imagem contribuem para o reconhecimento da infiltração amiloide cardíaca, pois avaliam a presença e a gravidade da hipertrofia ventricular e da disfunção sistólica e diastólica.^24-26^ No entanto, as alterações morfológicas e funcionais mais típicas são observadas em estágios mais avançados da doença e correlacionam-se com a quantidade de depósitos amiloide sistêmico e com piora nos sinais e sintomas clínicos.^27,28^

Entre os achados ecocardiográficos em nossa casuística, observamos que os pacientes com ATTRw apresentavam-se com maior grau de disfunção diastólica que o ATTRh, o que pode ser justificado pelo maior tempo de doença e pela maior prevalência de sintomas.

Quando comparamos as formas de amiloidose cardíaca, é descrito que pacientes com forma ATTRw caracterizam-se por maior hipertrofia ventricular esquerda, menor fração de ejeção e o *strain* longitudinal é menor nas formas ATTRw e AL do que nas formas ATTRh.^2^ Em nossa casuística, não encontramos diferenças significativas entre os tipos no que diz respeito à hipertrofia ou função. Os pacientes com ATTRw e maior tempo de doença apresentavam maiores diâmetros diastólicos do ventrículo esquerdo e de átrio esquerdo (p = 0,012 e p = 0,001 respectivamente).

Técnicas mais avançadas do ECO, como o s*train* e o s*train rate* derivados do *speckle tracking*, podem auxiliar na avaliação de movimentos cardíacos de torção e facilitar a diferenciação entre amiloidose cardíaca e miocardiopatia hipertrófica, porém ainda não são realizadas rotineiramente na prática clínica.^24^ O achado sugere a necessidade de complementação diagnóstica com outros métodos de imagem, tais como RMC e cintilografia óssea.

A RMC é outro método de avaliação por imagem que fornece informações sobre a função e a morfologia cardíaca em pacientes com amiloidose.^25,26^ O realce tardio de padrão mais específico é o subendocárdico difuso e circunferencial do ventrículo esquerdo.^29,30^ Em nosso trabalho, obtivemos informações sobre a presença de realce tardio. No entanto, devido à falta de padronização, não foi possível realizar a avaliação segmentar. Todavia, dos 55 pacientes com essa informação, 71% apresentavam presença de realce tardio de padrão não isquêmico.

O padrão assimétrico de hipertrofia miocárdica nos pacientes com amiloidose ATTR difere dos indivíduos com forma AL, geralmente simétrico. Em estudo de 263 pacientes com amiloidose ATTR, confirmada pela cintilografia com PYP-^99m^Tc, comparados com 50 indivíduos com a forma AL, observou-se na forma ATTR a presença de hipertrofia assimétrica em 79% dos casos, simétrica em 18% e ausente em 3% dos casos. O padrão de realce tardio foi 29% subendocárdico e 71% transmural.^27^ Em nossa casuística, observamos menor incidência de assimetria na hipertrofia na forma ATTR e maior incidência do que a esperada na forma AL.

Em 2016, Gillmore et al. demonstraram que a cintilografia miocárdica com PYP-^99m^Tc permite o diagnóstico confiável de amiloidose ATTR, sem a necessidade de confirmação histológica (biopsia cardíaca) nos pacientes que não apresentam picos monoclonais.^31^ Em nossa coorte, os dados sobre a cintilografia cardíaca foram limitados, devido à heterogeneidade de protocolo inerente à implementação do método. Mesmo assim, pudemos observar que, conforme o trabalho de Gilmore, a forma AL apresenta captação com 3 horas, algo significativamente menor do que na ATTR. Atualmente, é o método validado com melhor sensibilidade e especificidade para amiloidose por transtirretina, quando afastadas as gamopatias monoclonais.^32^

Como já descrito por outros autores, observamos a amiloidose como um grande mimetizador de outras formas de doenças cardíacas. Ela se manifesta tanto na forma de insuficiência cardíaca, com fração de ejeção reduzida (quadros mais avançados) preservada (ICFEp), na forma de cardiomiopatia hipertrófica (simétrica ou assimétrica), quanto na forma de arritmias atriais, ventriculares e distúrbios do sistema de condução, simulando outras doenças e dificultando seu diagnóstico.^11,12^

Observaram-se dados importantes, como o achado de que a mutação mais prevalente e associada ao acometimento cardiológico em nosso meio foi a Val142Ile, a despeito do esperado pela colonização portuguesa, na qual predomina a Val50Met como maior tempo para o diagnostico da forma ATTRw em nosso meio. Devemos ressaltar também a importância do reconhecimento dos sinais de alerta, como a dosagem dos biomarcadores para detectar o acometimento cardíaco na amiloidose; a presença de polineuropatia em pacientes com ICFEp e/ou cardiomiopatia hipertrófica; a gama de alterações eletrocardiográficas possíveis e a alta prevalência de arritmias nessa população (sobretudo a fibrilação atrial, com todas as suas implicações). Além disso, observamos a importância dos métodos de imagens, como marcadores de acometimento cardíaco através do *strain* longitudinal global do ventrículo esquerdo, captação após 3 horas na cintilografia com pirofosfato e presença do realce tardio na RMC.

### Limitações

Esse registro foi um estudo unicêntrico retrospectivo de uma amostra de conveniência, realizado em um serviço de referência cardiológico, com parte dos pacientes encaminhados de outros serviços, no qual nem sempre todos apresentavam a mesma padronização para a realização de alguns exames.

## Conclusões

A amiloidose é uma doença de apresentação fenotípica heterogênea, cujo diagnóstico precoce exige alto índice de suspeição clínica, com longo tempo entre início dos sintomas e sua constatação. Os biomarcadores, o eletrocardiograma e os métodos de imagens são fundamentais na investigação, sobretudo quando associados à história clínica sugestiva, como a polineuropatia concomitante à insuficiência cardíaca com fração de ejeção preservada e a pesquisa genética específica.

## References

[1] Benson MD, Buxbaum JN, Eisenberg DS, Merlini G, Saraiva MJM, Sekijima Y, et al. Amyloid nomenclature 2018: recommendations by the International Society of Amyloidosis (ISA) nomenclature committee. Amyloid. 2018;25(4):215-9. 10.1080/13506129.2018.1549825 30614283

[2] Quarta CC, Solomon SD, Uraizee I, Kruger J, Longhi S, Ferlito M, et al. Left ventricular structure and function in transthyretin-related versus light-chain cardiac amyloidosis. Circulation. 2014;129(18):1840-9. 10.1161/CIRCULATIONAHA.113.006242 24563469

[3] Pereira NL, Grogan M, Dec GW. Spectrum of restrictive and infiltrative cardiomyopathies: part 1 of a 2-part series. J Am Coll Cardiol. 2018;71(10):1130-48. 10.1016/j.jacc.2018.01.016 29519355

[4] Ando Y, Coelho T, Berk JL, Cruz MW, Ericzon BG, Ikeda S, et al. Guideline of transthyretin-related hereditary amyloidosis for clinicians. Orphanet J Rare Dis. 2013 Feb 20;8(1):1-18. 10.1186/1750-1172-8-31 PMC3584981 23425518

[5] Lousada I, Comenzo RL, Landau H, Guthrie S, Merlini G. Light chain amyloidosis: patient experience survey from the Amyloidosis Research Consortium. Adv Ther. 2015;32(10):920-8. 10.1007/s12325-015-0250-0 PMC4635176 26498944

[6] Falk RH, Alexander KM, Liao R, Dorbala S. AL (light-chain) cardiac amyloidosis: a review of diagnosis and therapy. J Am Coll Cardiol. 2016;68(12):1323-41. 10.1016/j.jacc.2016.06.053 27634125

[7] Perugini E, Guidalotti PL, Salvi F, Cooke RMT, Pettinato C, Riva L, et al. Noninvasive etiologic diagnosis of cardiac amyloidosis using 99mTc-3, 3-diphosphono-1, 2-propanodicarboxylic acid scintigraphy. J Am Coll Cardiol. 2005;46(6):1076-84. 10.1016/j.jacc.2005.05.073 16168294

[8] Dorbala S, Ando Y, Bokhari S, Dispenzieri A, Falk RH, Ferrari VA, et al. ASNC/AHA/ASE/EANM/HFSA/ISA/SCMR/SNMMI expert consensus recommendations for multimodality imaging in cardiac amyloidosis: Part 1 of 2 – evidence base and standardized methods of imaging. J Nucl Cardiol. 2019;26(6):2065-123. 10.1007/s12350-019-01760-6 31468376

[9] Brandão SCS, Quagliato PC, Lopes RW, Matushita CS, Amorin BJ, Mesquita CT. Guideline de cintilografia com marcadores ósseos para pesquisa de amiloidose cardíaca por transtirretina [citado 5 maio 2021]. Disponível em: https://sbmn.org.br/wp-content/uploads/2019/10/Guideline-Cintilografia-com-Pirofosfato-Tc99m-Amiloidose-10.2019.pdf.

[10] Cruz MW, Pinto MV, Pinto LF, Gervais R, Dias M, Perez C, et al. Baseline disease characteristics in Brazilian patients enrolled in transthyretin amyloidosis outcome survey (THAOS). Arq NeuroPsiquiatr. 2019;77(2):96-100. 10.1590/0004-282X20180156 30810593

[11] Pagourelias ED, Mirea O, Duchenne J, Cleemput JV, Delforge M, Bogaert J, et al. Echo parameters for differential diagnosis in cardiac amyloidosis: a head-to-head comparison of deformation and nondeformation parameters. Circ Cardiovasc Imaging. 2017;10(3):e005588. 10.1161/CIRCIMAGING.116.005588 28298286

[12] Ruberg FL, Grogan M, Hanna M, Kelly JW, Maurer MS. Transthyretin amyloid cardiomyopathy: JACC state-of-the-art review. J Am Coll Cardiol. 2019;73(22):2872-91. 10.1016/j.jacc.2019.04.003 PMC6724183 31171094

[13] Nagueh SF, Appleton CP, Gillebert TC, Marino PN, Oh JK, Smiseth OA et al. Recommendations for the evaluation of left ventricular diastolic function by echocardiography. Eur J Echocardiogr. 2009;10(2):165-93. 10.1093/ejechocard/jep007 19270053

[14] Sekijima Y, Ueda M, Koike H, Misawa S, Ishii T, Ando Y. Diagnosis and management of transthyretin familial amyloid polyneuropathy in Japan: red-flag symptom clusters and treatment algorithm. Orphanet J Rare Dis. 2018;13(1):6. 10.1186/s13023-017-0726-x PMC5773042 29343286

[15] Queiroz MCC, Pedrosa RC, Berensztejn AC, Pereira BB, Nascimento EM, Duarte MMT, et al. Frequency of cardiovascular involvement in familial amyloidotic polyneuropathy in brazilian patients. Arq Bras Cardiol. 2015;105(5):503-9. 10.5935/abc.20150112 PMC4651409 26351985

[16] Sattianayagam PT, Hahn AF, Whelan CJ, Gibbs SDJ, Pinney JH, Stangou AJ, et al. Cardiac phenotype and clinical outcome of familial amyloid polyneuropathy associated with transthyretin alanine 60 variant. Eur Heart J. 2012;33(9):1120-7. 10.1093/eurheartj/ehr383 21992998

[17] Maurer MS, Elliott P, Comenzo R, Semigran M, Rapezzi C. Addressing common questions encountered in the diagnosis and management of cardiac amyloidosis. Circulation. 2017;135(14):1357-77. 10.1161/CIRCULATIONAHA.116.024438 PMC5392416 28373528

[18] Cruz MW, Foguel D, Berensztejn A, Pedrosa R, Silva PF. The phenotypical expression of an European inherited TTR amyloidosis in Brazil. Orphanet J Rare Dis. 2015;10(Suppl 1):O7.

[19] Cheung CC, Roston TM, Andrade JG, Bennett MT, Davis MK. Arrhythmias in cardiac amyloidosis: challenges in risk stratification and treatment. Can J Cardiol. 2020;36(3):416-23. 10.1016/j.cjca.2019.11.039 32145868

[20] Zoni-Berisso M, Lercari F, Carazza T, Domenicucci S. Epidemiology of atrial fibrillation: European perspective. Clin Epidemiol. 2014 Jun 16;6:213-20. 10.2147/CLEP.S47385 PMC4064952 24966695

[21] Lippi G, Sanchis-Gomar F, Cervellin G. Global epidemiology of atrial fibrillation: an increasing epidemic and public health challenge. Int J Stroke. 2021;16(2):217-21. 10.1177/1747493019897870 31955707

[22] Donnellan E, Wazni OM, Hanna M, Elshazly MB, Puri R, Saliba W, et al. Atrial fibrillation in transthyretin cardiac amyloidosis: predictors, prevalence and efficacy of rhythm control strategies. JACC Clin Electrophysiol. 2020;6(9):1118-27. 10.1016/j.jacep.2020.04.019 32972546

[23] Khanna S, Lo P, Cho K, Subbiah R. Ventricular arrhythmias in cardiac amyloidosis: a review of current literature. Clin Med Insights Cardiol. 2020 Sep 29;14:1179546820963055. 10.1177/1179546820963055 PMC7545745 33088185

[24] Phelan D, Collier P, Thavendiranathan P, Popovic ZB, Hanna M, Plana JC, et al. Relative apical sparing of longitudinal strain using two-dimensional speckle-tracking echocardiography is both sensitive and specific for the diagnosis of cardiac amyloidosis. Heart. 2012;98(19):1442-8. 10.1136/heartjnl-2012-302353 22865865

[25] Fontana M, Pica S, Reant P, Abdel-Gadir A, Treibel TA, Banypersad SM, et al. Prognostic value of late gadolinium enhancement cardiovascular magnetic resonance in cardiac amyloidosis. Circulation. 2015;132(16):1570-9. 10.1161/CIRCULATIONAHA.115.016567 PMC4606985 26362631

[26] Martinez-Naharro A, Treibel TA, Abdel-Gadir A, Bulluck H, Zumbo G, Knight DS, et al. Magnetic resonance in transthyretin cardiac amyloidosis. J Am Coll Cardiol. 2017;70(4):466-77. 10.1016/j.jacc.2017.05.053 28728692

[27] Clesham GJ, Vigushin DM, Hawkins PN, Pepys MB, Oakley CM, Nihoyannopoulos P. Echocardiographic assessment of cardiac involvement in systemic AL amyloidosis in relation to whole body amyloid load measured by serum amyloid P component (SAP) clearance. Am J Cardiol. 1997;80(8):1104-8. 10.1016/s0002-9149(97)00617-6 9352992

[28] Hongo M, Ikeda S. Echocardiographic assessment of the evolution of amyloid heart disease: a study with familial amyloid polyneuropathy. Circulation. 1986;73(2):249-56. 10.1161/01.cir.73.2.249 3943160

[29] Syed IS, Glockner JF, Feng D, Araoz PA, Martinez MW, Edwards WD, et al. Role of cardiac magnetic resonance imaging in the detection of cardiac amyloidosis. JACC Cardiovasc Imaging. 2010;3(2):155-64. 10.1016/j.jcmg.2009.09.023 20159642

[30] Baroni M, Nava S, Quattrocchi G, Milazzo A, Giannattasio C, Roghi A, et al. Role of cardiovascular magnetic resonance in suspected cardiac amyloidosis: late gadolinium enhancement pattern as mortality predictor. Neth Heart J. 2018;26(1):34-40. 10.1007/s12471-017-1046-4 PMC5758446 29058206

[31] Gillmore JD, Maurer MS, Falk RH, Merlini G, Damy T, Dispenzieri A, et al. Nonbiopsy diagnosis of cardiac transthyretin amyloidosis. Circulation. 2016;133(24):2404-12. 10.1161/CIRCULATIONAHA.116.021612 27143678

[32] Sperry BW, Reyes BA, Ikram A, Donnely JP, Phelan D, Jaber WA, et al. Tenosynovial and cardiac amyloidosis in patients undergoing carpal tunnel release. J Am Coll Cardiol. 2018;72(17):2040-50. 10.1016/j.jacc.2018.07.092 30336828

