# Supplementary material for: Perfil Clínico, Laboratorial e de Métodos de Imagem na Amiloidose Sistêmica em um Centro de Referência Cardiológico Brasileiro
Source: Arq Bras Cardiol. 2021 Dec 20;118(2):422–32. [Article in Portuguese] doi: 10.36660/abc.20201003 (PMC8856672; doi:10.36660/abc.20201003)
Supplement: Translation

# Translation: Clinical, Laboratory, and Imaging Profile in Patients with Systemic Amyloidosis in a Brazilian Cardiology Referral Center

**Authors:** Fábio Fernandes, Aristóteles Comte de Alencar, Bruno Vaz Kerges Bueno, Caio Rebouças Fonseca Cafezeiro, João Henrique Rissato, Roberta Shcolnik Szor, Mariana Lombardi Peres de Carvalho, Wilson Mathias, Angelina Maria Martins Lino, Jussara Bianchi Castelli, Evandro de Oliveira Souza, Félix José Alvarez Ramires, Viviane Tiemi Hotta, José Soares, Caio de Assis Moura Tavares, José Eduardo Krieger, Carlos Eduardo Rochitte, André Dabarian, Ludhmila Abrahão Hajjar, Roberto Kalil, Charles Mady

**Affiliations:** 1 Brazil Instituto do Coração do Hospital das Clínicas da Faculdade de Medicina da Universidade de São Paulo, São Paulo, SP – Brazil; 2 Brazil Hospital das Clínicas da Faculdade de Medicina da Universidade de São Paulo, São Paulo, SP – Brazil; 3 Brazil Universidade de São Paulo - Instituto do Câncer de São Paulo Octavio Frias de Oliveira, São Paulo, SP – Brazil

**Keywords:** Amyloidosis, Immunoglobulin Light Chains, Heart Failure, Prealbumin, Hypertrophic Cardiomyopathy, Diagnosis, Imaging

## Abstract

**Background:**

Systemic amyloidosis is a disease with heterogeneous clinical manifestations. Diagnosis depends on clinical suspicion combined with specific complementary methods.

**Objective:**

To describe the clinical, laboratory, electrocardiographic, and imaging profile in patients with systemic amyloidosis with cardiac involvement.

**Methods:**

This study was conducted with a convenience sample, analyzing clinical, laboratory, electrocardiographic, echocardiographic, nuclear medicine, and magnetic resonance data. Statistical significance was set at p < 0.05.

**Results:**

A total of 105 patients were evaluated (median age of 66 years), 62 of whom were male. Of all patients, 83 had transthyretin (ATTR) amyloidosis, and 22 had light chain (AL) amyloidosis. With respect to ATTR cases, 68.7% were the hereditary form (ATTRh), and 31.3% were wild type (ATTRw). The most prevalent mutations were Val142Ile (45.6%) and Val50Met (40.3%). Time from onset of symptoms to diagnosis was 0.54 and 2.15 years, in the AL and ATTR forms, respectively (p < 0.001). Cardiac involvement was observed in 77.9% of patients with ATTR and in 90.9% of those with AL. Alterations were observed in atrioventricular and intraventricular conduction in 20% and 27.6% of patients, respectively, with 33.7% in ATTR and 4.5% in AL (p = 0.006). In the ATTRw form, there were more atrial arrhythmias than in ATTRh (61.5% versus 22.8%; p = 0.001). On echocardiogram, median septum thickness in ATTRw, ATTRh, and AL was 15 mm, 12 mm, and 11 mm, respectively (p = 0.193). Elevated BNP was observed in 89.5% of patients (median 249, ICR 597.7), and elevated troponin was observed in 43.2%.

**Conclusion:**

In this setting, it was possible to characterize cardiac involvement in systemic amyloidosis in its different subtypes by means of clinical history and the diagnostic methods described.

## Introduction

Amyloidosis refers to a set of rare diseases where protein fragments, folded in highly stable aggregates (beta-pleated sheets), are pathogenically deposited in the extracellular space of organs and tissues, in the form of insoluble fibrils. Depending on the type of subunit polymers (monomers) of protein deposits, there are different disease subtypes.

Cardiac involvement is frequent; it manifests with thickening and structural breakdown, and it can cause diastolic and systolic dysfunction, heart failure, conduction disorders, and atrial and ventricular arrhythmias, with high morbidity and mortality. Of all known forms of amyloidosis (36 to date), the majority of cases of cardiac amyloidosis are due to deposition of 2 proteins: light chains (AL) or transthyretin (ATTR).^1-3^

The most common systemic form is caused by deposits of light chains, which refer to clones of these chains associated with antibodies formed by plasma cell clones in bone marrow (AL amyloidosis). Transthyretin, on the other hand, is a protein produced in the liver, and it carries thyroxine and retinol. Its monomeric form is more prone to misfolding and depositing in tissues, thus generating amyloidosis.

There are 2 main subtypes of ATTR amyloidosis: wild-type amyloidosis (ATTRw), formerly known as “senile,” where, over the years, fragments of a normally produced protein accumulate in the tissues; and mutant or hereditary ATTR (ATTRh), were patients carry pathological mutations in the transthyretin gene that predispose them to accelerated deposition of these proteins, with more than 120 mutations described to date.^1,3^

Establishing diagnosis of cardiac amyloidosis is difficult, and it requires a high degree of clinical suspicion. In this context, imaging exams, especially echocardiography, scintigraphy, and cardiac magnetic resonance, have increasingly contributed to the recognition of amyloid deposits.

Correct diagnosis is necessary, because the usual treatment for heart failure cannot always be applied in amyloidotic cardiomyopathy; prognosis is different depending on the etiology; the evolution and treatment are different from the other hypertrophic cardiomyopathies, and there are currently specific therapeutic possibilities, which can modify the natural history of the disease. Moreover, it is important to note that diagnosis of AL amyloidosis constitutes a cardiac emergency.

Studies have shown that diagnosis of this systemic disease generally occurs in a delayed fashion, with the time from onset of symptoms until establishment of diagnosis taking 2 years in the AL form and up to 4 years in the ATTR form. Approximately 32% of patients were attended by up to 5 different doctors before receiving diagnosis, and 39% already presented limiting neuropathy that compromised treatment of the disease.^4,5^ There are, however, no specific data regarding time of onset of cardiovascular symptoms and disease diagnosis in the Brazilian population.

## Objective

To describe clinical, laboratory, electrocardiographic, and imaging profile of a group of patients with systemic amyloidosis referred to a cardiology referral center, in order to better understand the characteristics of the disease in Brazil, promoting the creation of new management strategies.

## Methods

### Design and Study Population

This study included patients followed up at our service, who had confirmed diagnosis of systemic amyloidosis with cardiomyopathy, and individuals referred from other health units of different specialties (neurology, hematology, nephrology, and gastroenterology) for evaluation of cardiac involvement (amyloidotic cardiomyopathy), after the disease had already been confirmed in other organs and systems. During outpatient follow-up, patients underwent investigation of amyloidosis, in accordance with the current, adopted, international flowchart (Figure 1),^6^ carrying out electrocardiogram (ECG), echocardiogram, bone scintigraphy with PYP-^99m^Tc, cardiac magnetic resonance, protein electrophoresis with immunofixation, free light chain assay, and genetic investigation for transthyretin mutations. Genotype screening of family members of patients with hereditary amyloidosis was carried out.

**Figure 1:** – Flowchart for diagnosis of cardiac amyloidosis (adapted from reference 8). AL: light chain amyloidosis; ATTR: transthyretin amyloidosis; ATTRh: transthyretin amyloidosis, hereditary form; ATTRw: transthyretin amyloidosis, wild type; ECG: electrocardiogram.

Data were collected during the period from January 2018 to May 2020.

### Inclusion and Exclusion Criteria

This sample included patients with clinical, laboratory, and imaging phenotype, with suspicion of amyloidosis, who were referred for evaluation or followed up at our service.

The following were excluded: patients without proven amyloidosis, using the methods described; patients awaiting supporting diagnostic tests; and patients with other forms of heart disease, such as hypertensive, ischemic, Chagas, or concomitant valve disease (aortic stenosis).

### Diagnostic Criteria

Cases of transthyretin amyloidosis were defined by the absence of monoclonal component identified by electrophoresis with immunofixation of serum and urinary proteins, with normal serum free light chain assay and ratios (*kappa* and *lambda*), as well as the presence of Perugini grade II or III myocardial uptake on myocardial scintigraphy using pyrophosphate labeled with metastable technetium-99 (PYP-^99m^Tc).^7^ In cases of the AL form, diagnosis of amyloidosis was confirmed histologically by biopsy of abdominal fat, bone marrow, gingiva, or myocardium, once the monoclonal component was initially proven by one of the methods described above, in addition to absent myocardial uptake or grade I on scintigraphy.

### Imaging Methods

Echocardiography was performed using GE Healthcare Vivid E95 equipment (Waukesha, Wisconsin, USA), with evaluations in 2- and 3-dimensional mode in real time, with analysis of cardiac flows by Doppler echocardiography, tissue Doppler analysis, 2-dimensional speckle tracking analysis of left ventricular global longitudinal strain, and right ventricular strain analysis.

Images were acquired in parasternal longitudinal projections of left chambers, transverse, and apical 2-, 3-, and 4-chamber views, in accordance with the standards of the American Society of Echocardiography. Real-time 3-dimensional images were acquired during expiratory apnea with observation of the cardiac cycle based on the ECG record.

Myocardial scintigraphy with PYP-^99m^Tc was conducted with images obtained 1 and 3 hours after tracer injection, using a low-energy, high-resolution collimator. Anterior projection (1 hour and 3 hours) and static anterior, left lateral and left anterior oblique projections (3 hours) were used. When there was uptake in the cardiac area, SPECT images of the chest were performed, 3 hours after injection, in order to better identify the cardiac limits and prevent the “pool” from interfering with image interpretation, which would result in false positive results. Two image interpretation criteria were used, namely, visual and semi-quantitative analysis. The visual criterion was based on the Perugini scale, performing qualitative analysis of the 3-hour images by comparing the degree of cardiac concentration of the tracer with the degree of bone uptake in the ribcage. In grade 2, concentration is similar in both points evaluated, whereas, in grade 3, cardiac uptake is greater than the uptake observed in the ribs; both grades are strongly associated with the diagnosis of cardiac transthyretin amyloidosis. In the absence of uptake (grade 0), it was considered that there was no cardiac amyloid deposition; in the event of mild cardiac uptake that was lower than in the ribs (grade 1), the possibilities of amyloid deposition of light chains (AL) or initial phase of transthyretin deposition were considered. Semi-quantitative analyses of the degree of radiotracer uptake were performed, comparing the regions of interest in the cardiac area and the mirror area in the right hemithorax, in the previous image. Heart-to-contralateral ratio values of ≥ 1.5 on 1-hour images and ≥ 1.3 on 3-hour images were considered strongly suggestive of transthyretin amyloidosis. The criteria used are in accordance with the protocol of the American Society of Nuclear Cardiology and the guidelines for scintigraphy with bone markers of the Brazilian Society of Nuclear Medicine.^8,9^

Cardiac magnetic resonance was carried out using 1.5 Tesla devices (Achieva, Philips Medical Systems, Best, The Netherlands Signa CV/I; GE Medical Systems, Waukesha, WI, USA; and Avanto, Siemens Medical Solutions, Erlangen, Germany), with images obtained in short and long axes during apnea, with pulse sequences synchronized with the ECG. The first gradient-echo sequence was a steady-state free precession to evaluate left and right ventricular morphology and functions. The second sequence was a segmented gradient-echo with an inversion-recovery pulse to obtain delayed myocardial enhancement 10 to 20 minutes after intravenous administration of 0.2 mmol/kg of gadolinium-based contrast agent (Dotarem®, gadoteric acid, Gd-DOTA, Guerbet Aulnay-Sous-Bois, France). For cine imaging, using steady-state free precession sequences, the following parameters were applied: repetition time, 3.4 ms; echo time, 2.0 ms; deflection angle, 45º; matrix, 256 × 160; cardiac phases,^20^ views per segment, 8 to 16 to obtain temporal resolution of 55 ms or less; slice thickness, 8 mm; interslice gap, 2 mm; and field of view, 36 to 40 cm. With respect to pulse sequence of delayed myocardial enhancement, the following parameters were applied in the short and long axes: repetition time, 7.3 ms; echo time, 3.2 ms; deflection angle, 25º; matrix, 256 × 196; slice thickness, 8 mm; interslice gap, 2 mm; field of view, 36 to 40 cm; inversion time, 200 ms to 300 ms; receiving bandwidth, 32.5 kHz; every RR acquisition and number of excitations, 2. Short-axis views were set from the base to the apex (in general 8 to 12 cine-slices/heart) perpendicular to the long ventricular axis, covering the entire left ventricle. It is important to note that slice locations were the exact same for both pulse sequences, making it possible to compare function and morphology with the tissue characterization provided by delayed myocardial enhancement.

### Variables Analyzed

This study evaluated the following: age; sex; time from onset of symptoms to confirmation of diagnosis; initially affected system (cardiological, neurological, both, or others); ECG and/or 24-hour Holter alterations (heart rate, low voltage, chamber overloads, atrioventricular or intraventricular conduction disorders, arrhythmias); transthoracic echocardiogram data (interventricular septum thickness, left ventricular ejection fraction measured by Simpson’s method, diastole pattern, presence or absence of valve alteration, apical sparing pattern); cardiac magnetic resonance data; data from scintigraphy with PYP-^99m^Tc (degree of marker uptake and ratio of uptake between the cardiac region and the right thoracic region after 1 and 3 hours); BNP or NT-pro-BNP values; elevated serum troponin; presence or absence of monoclonal components in immunofixation of serum and urinary proteins; serum free light chain assay; and the ratio between the former 2 variables.

The following cardiovascular symptoms were considered: palpitations, chest pain, symptomatic hypotension, orthostatic hypotension, syncope, dyspnea on exertion, and orthopnea associated with lower limb edema; heart failure was defined when the following were observed: pathological jugular turgescence, hepatic-jugular reflux, paroxysmal nocturnal dyspnea, pulmonary rales, third heart sound, bilateral lower limb edema, and dyspnea on exertion.

### Statistical analysis

The sample size used in this study was by convenience sampling. Continuous variables with normal distribution were described as mean and standard deviation, and continuous variables without normal distribution were described only as median and interquartile range.

The Mann-Whitney test was used for comparison between the quantitative variables of age, duration of clinical disease, septum thickness on echocardiogram, left ventricular ejection fraction, uptake values on scintigraphy with PYP-^99m^Tc, and serum levels of BNP and NT-pro-BNP. We tested the normality of the quantitative main outcome variables using the Kolmogorov-Smirnov test, and we concluded that there was no guaranteed distribution of normality.

The chi-square test was applied for analysis of statistical dependence and frequency distribution of the qualitative variables of sex; ECG and 24-hour Holter alterations; presence or absence of monoclonal peaks on immunofixation; alterations in valves, septum thickness, and diastole on echocardiogram; presence or absence of late enhancement on resonance; and troponin elevation.

In order to compare the proportion of responses between 2 variables, the two-proportion equality test was used, and the comparison between the AL, ATTRh, and ATTRw groups for quantitative variables was performed using the Kruskal-Wallis test.

The statistical software SPSS, version 20 (IBM Corp., Armonk, NY, USA) and Minitab 16 (Minitab, LLC) were used for analyses. All tests were considered statistically significant if a p value < 0.05 was found.

The project received approval from the Scientific and Ethics Committee of the Heart Institute of the *Hospital das Clínicas* of the Faculty of Medicine of the University of São Paulo (InCor, HC-FMUSP) and the Ethics Committee for Analysis of Research Projects (CAPPesq), of the Clinical Board of the *Hospital das Clínicas* of the University of São Paulo (CAAE number 27437019.5.0000.0068.)

## Results

### Epidemiological Profile

This study evaluated 105 patients, with median age of 66 years. Median age was 64 years in patients with the ATTR form and 66 years in those with the AL form. When evaluating subtypes of transthyretin amyloidosis, median age in the ATTRh form was 56 years, while it was 79 years in the ATTRw form.

In our study, 62 patients (59%) were male, and 54 of them had the ATTR form (34 ATTRh and 20 ATTRw), while 8 had the AL form. Of the 43 female patients, 29 had the ATTR form (23 ATTRh and 6 ATTRw), and 14 had the AL form.

### Types of amyloidosis

The most prevalent subtype of amyloidosis was secondary to transthyretin mutation, with the hereditary form (ATTRh) being the most common. Five different mutations were observed; Val142Ile (replacement of the amino acid valine at position 142 by isoleucine) was the most frequent, followed by Val50Met (replacement of the amino acid valine at position 50 by methionine) (Figure 2).

**Figure 2:** – Distribution of mutations found in ATTRh. Ala39Asp: substitution of alanine by aspartate at position 39; Phe84Leu: substitution of phenylalanine by leucine at position 84; Thr80Al: substitution of threonine by alanine at position 80; Val142Ile: substitution of valine by isoleucine at position 142; Val50Met: substitution of valine by methionine at position 50.

### Time to diagnosis

Average time from onset of symptoms to diagnosis was 0.54 ± 1.94 years in the AL form and 2.15 ± 2.43 years in the ATTR form (p < 0.001). In the ATTRh subtype, average time was 16 months, and, in ATTRw, it was 37 months (p < 0.049).

### Clinical Presentation

Clinical presentation at the first consultation consisted mainly of cardiological symptoms, either alone or associated with neurological symptoms (peripheral polyneuropathy, altered gastrointestinal habits, carpal tunnel syndrome, bladder alterations), regardless of the form of amyloidosis (Table 1)

**Table 1:** – Clinical presentation in different subtypes of amyloidosis

Systems affected	AL		ATTR		Total	
	N	%	N	%	N	%
Asymptomatic	3	15.80%	14	18.70%	17	18.10%
Cardiovascular	15	78.90%	41	54.70%	56	59.60%
Neurological	1	5.30%	15	20.00%	16	17.00%
Mixed	0	0.00%	5	6.70%	5	5.30%

*Total number of patients: 105; missing initial data: 11; p = 0.189; AL: light chain amyloidosis; ATTR: transthyretin amyloidosis*

It was observed that 31% of our patients had an association of painful neuropathy, gait disturbances, bilateral carpal tunnel syndrome, or autonomic dysfunction with ventricular hypertrophy and/or heart failure with preserved ejection fraction.

In our study, 17 patients were asymptomatic. This occurred due to screening of family members of probands with ATTRh. The most prevalent symptom was dyspnea alone (38%) or dyspnea associated with other symptoms (urinary incontinence, polyneuropathy, dizziness, autonomic dysfunction, syncope) in 16%. In 5% of cases, the first symptom was palpitations, which was generally related to subsequent diagnosis of cardiac arrhythmia.

### Biomarkers

Increased serum BNP levels were observed in 94 patients (89.5%) (median 249 ng/ml, IQR 597.7), and the increase was greater in patients with ATTR than in those with AL.

Serum troponin levels were above the upper limit of normal in 43.2% of the patients studied.

Electrophoresis of serum or urinary proteins showed monoclonal peaks in only 3 patients with ATTR, which were considered as gammopathy of uncertain value, after they were subjected to hematological evaluation and bone marrow biopsy.

### Twelve-lead Electrocardiogram and 24-hour Holter

More than half of the patients showed some type of ECG abnormality. It was observed that one fifth of the patients had non-sinus rhythm on ECG, atrial fibrillation being the most commonly found. The other rhythms observed were atrial flutter, atrial ectopic rhythm, and pacemaker rhythm (Tables 2 and 3).

**Table 2:** – ECG alterations in different types of amyloidosis

Findings	Altered ECG	AVB	IVB	Atrial arrhythmia	Ventricular arrhythmia	Atrial fibrillation
Total	72 (68.6%)	21 (20%)	29 (27.6%)	36 (34.3%)	11 (10.5%)	16 (15.2%)
AL	14 (66.7%)	2 (9.1%)	1 (4.5%)	7 (31.8%)	3 (13.6%)	2 (9.1%)
ATTR	58 (80.6%)	19 (22.9%)	28 (33.7%)	29 (34.9%)	8 (9.6%)	14 (16.9%)
p	0.180	0.150	0.006	0.784	0.586	0.719

*AL: light chain amyloidosis; ATTR: transthyretin amyloidosis; AVB: atrioventricular block; ECG: electrocardiogram; IVB: intraventricular block. Test method: chi-square.*

**Table 3:** – ECG alterations in subtypes of ATTR

Findings	Altered ECG n (%)	AVB n (%)	IVB n (%)	Atrial arrhythmia n (%)	Ventricular arrhythmia n (%)	Atrial fibrillation n (%)
ATTR	58 (80.6)	19 (22.9)	28 (33.7)	29 (34.9)	8 (9.6)	14 (16.9)
ATTRh	36 (76.6)	11 (19.3)	17 (29.8)	13 (22.8)	4 (7)	4 (7)
ATTRw	22 (88)	8 (30.8)	11 (42.3)	16 (61.5)	4 (15.4)	10 (38.5)
p	0.244	0.249	0.265	0.001	0.231	0.003

*ATTR: transthyretin amyloidosis; ATTRh: transthyretin amyloidosis, hereditary form; ATTRw: transthyretin amyloidosis, wild type; AVB: atrioventricular block; ECG: electrocardiogram; IVB: intraventricular block. Test method: chi-square.*

In addition to sustained arrhythmias, atrial extrasystole and paroxysmal atrial tachycardia were also observed on Holter in 12 patients, and ventricular extrasystole, accelerated idioventricular rhythm, and nonsustained ventricular tachycardia were observed in 14 patients, with overlapping arrhythmia in some.

Conduction disorders were found in almost half of the patients. The most commonly found alteration in atrioventricular conduction was first-degree block, and 5 patients required cardiac pacemaker implantation. When evaluating intraventricular conduction (bundle-branch blocks and divisional blocks), it was observed that conduction disorder was much more frequent in the ATTR form of amyloidosis than in the AL form (Tables 2 and 3). In 20 patients (19.1%), low voltage was observed (QRS amplitude < 5 mm in the limb leads or < 10 mm in the precordial leads), and 21 (20%) had a pseudo-infarct pattern on ECG (defined as the presence of pathological Q waves in at least two contiguous ECG leads, without obstructive coronary disease).

### Echocardiogram

Absolute values of left ventricular ejection fraction were higher in patients with the AL form than in those with the ATTR form, but the difference between the types was not statistically significant (Table 4).

**Table 4:** – Echocardiographic parameters in subtypes of amyloidosis

Parameters	ATTRh Median (IQR)	ATTRw Median (IQR)	AL Median (IQR)	p
Septum (mm)	12 (7.3)	15 (6.5)	11 (4.0)	0.193
Posterior wall (mm)	11 (5.8)	12 (4.5)	11 (2.0)	0.531
Diastolic diameter (mm)	45 (5.5)	49.5 (13)	44 (8.0)	**0.012**
Systolic diameter (mm)	30 (6.0)	32 (14)	29 (7.0)	0.055
LVEF (%)	60.5 (26)	58.5 (19.3)	62 (16)	0.230
Left atrial diameter (mm)	42 (9.0)	45 (7.8)	38.5 (9.3)	**0.001**
PASP (mmHg)	32 (11.5)	34 (15)	33 (10.8)	0.813

*AL: light chain amyloidosis; ATTRh: transthyretin amyloidosis, hereditary form; ATTRw: transthyretin amyloidosis, wild type; IQR: interquartile range; LVEF: left ventricular ejection fraction; PASP: pulmonary artery systolic pressure. Analyses by Mann-Whitney test.*

Half of the patients showed thickening of the interventricular septum, and the pattern of asymmetric and symmetrical hypertrophy was found equally. When analyzing by amyloidosis subtype, more cases of asymmetric hypertrophy were observed in the ATTR form than in the AL form, but the difference was not statistically significant.

Both left ventricular diastolic diameter and left atrial diameter measurements were greater in patients with ATTRw than in those with ATTRh and AL, and the difference was statistically significant (Table 4). There was no difference regarding interventricular septum thickness, left ventricular systolic diameter, or pulmonary artery systolic pressure.

In the patients who underwent strain analysis (n = 22), apical sparing pattern was observed in 81.8%.

Approximately 53% of cases manifested symptoms of heart failure with preserved left ventricular ejection fraction.

Of the 16 patients who had exclusively neurological symptoms at the beginning of evaluation, 5 showed ventricular hypertrophy on echocardiogram.

### Magnetic Resonance

Of the 105 patients, 58 underwent magnetic resonance, and 24 (41%) presented left ventricular hypertrophy, with 17 cases in the ATTR form and 7 in the AL form. In the ATTR form, 10 were ATTRh, and 7 were ATTRw. The presence of late enhancement in a non-ischemic pattern was observed in 38 of these patients (68%), regardless of the presence of ventricular hypertrophy. Quantification of extracellular volume and T1 mapping were not carried out on a routine basis, it not being possible to conduct an analysis.

### Nuclear Medicine

Scintigraphy with PYP-^99m^Tc was performed in 66 patients. It was considered positive in 40 of them (60%), in accordance with the previously described criteria. It was observed that, in analysis after 3 hours, degree of uptake was significantly higher in patients with the ATTR form (median 1.54, IQR 0.42) than in those with the AL form (median 1.18, IQR 0.02) (p = 0.028). In the ATTRw subtype, median uptake was 1.6 (IQR 0.29), which was higher than in ATTRh, with median of 1.27 (IQR 0.6); however, this was not statistically significant (p = 0.044). In 12 patients with the ATTRh form, there was no uptake or only mild uptake, and, in 3 patients, the exam was considered inconclusive, because there was a discordant pattern between visual and semi-quantitative analyses.

Taking all of the evaluated cardiac variables into consideration, only 18 patients showed no alterations, namely, 2 patients with the AL form and 16 with the ATTRh form; the majority of these patients were evaluated because they were family members of index cases.

## Discussion

In our sample, we analyzed data acquired during the last 2 years, and we characterized 105 patients with amyloidosis. Cardiac involvement was present in 83% of these cases, even in the hereditary forms that do not affect the heart as a main point of focus (Val50Met, for example), corroborating data that demonstrate that the heart is a target organ of the disease.

According to a review of data in the literature, this is the Brazilian study with the largest sample of cardiology patients to date.

The largest published sample in Brazil, by Cruz et al., included 160 patients, but involvement was predominantly neurological, with 35.2% of the sample having cardiac involvement.^10^

In our center, the ATTRh form was the most common, followed by the wild type (ATTRw) and AL.^11^ Our sample differs from the international literature, where the most common form is AL. This finding may be explained by the fact that our center is located in a cardiology referral hospital,^12^ while patients with the AL form are referred for hematological follow-up in another institute within the same hospital complex.

Of all the mutations described in ATTRh to date, Val142Ile is the most common in the United States, where it is found in 3% to 4% of people who are African-American. It usually presents with symptoms of heart failure and ventricular hypertrophy around the seventh decade of life.^13^ In our study, this was also the most prevalent mutation, probably due to the fact that we are a cardiology referral service, demonstrating that it is more common than the late form of Val50Met.

Regarding cases of ATTR, it is currently known that the wild form is the most prevalent in the world, and it is commonly underdiagnosed.^12^ We believe that, despite advances in diagnosis of ATTR in our service, there are still a certain number of hidden cases, for instance, among patients diagnosed with aortic stenosis or hypertrophic cardiomyopathy. Two patients with the Val142Ile mutation were in the cohort of patients diagnosed with hypertrophic cardiomyopathy, and 1 of them had a homozygous mutation.

In Brazil and worldwide, the Val50Met mutation is the most prevalent.^14,15^ It sometimes has a bimodal pattern in some countries, and it presents a primarily neurological pattern when it affects younger patients, from 30 years of age, and a mixed neurological and cardiac pattern during the second peak in age, from 50 years of age.^14^ As we are a tertiary service, the pattern that we observed in patients with this mutation, the second most frequent in our study, was that of mixed involvement, from age 50 years.

The third most frequently observed mutation was Thr80Ala, which has been described in British and Irish patients, with onset of symptoms at around 60 years of age, showing predominantly cardiac and autonomic nerve phenotype, with less involvement of peripheral neuropathy and worse prognosis when compared to Val50Met.^16^

It is known that the wild form generally occurs later than the hereditary form. In our study, the mean age of ATTRw was 22 years greater than the hereditary form, denoting more elderly patients with more comorbidities, where, in general, other etiologies for cardiovascular symptoms or structural or electrophysiological alterations that are present are concluded in an early manner, without considering amyloidosis as an etiological possibility.

Time from symptom onset to diagnosis was longer in the ATTR form than in the AL form, possibly because the latter has a more pronounced clinical presentation, given that immunoglobulins present direct toxicity to cardiac tissue.^5^ When comparing the ATTR subtypes, ATTRw had a longer duration of disease until diagnosis than ATTRh, which can be explained by the fact that the hereditary form occurs in younger patients, and sometimes there has already been a diagnosed index case, which leads to family screening.

In patients with clinical complaints of heart failure, a little over half presented a pattern of heart failure with preserved ejection fraction, which is a frequent entity in clinical practice, especially in elderly patients, and it is usually considered only as a diastolic disorder related to age and associated comorbidities. This should be a warning sign for considering the disease, especially when it is associated with elevated levels of biomarkers.

Other signs and symptoms that should lead to diagnostic suspicion are the presence of sensory-motor polyneuropathy (painful neuropathy, gait disturbances, and bilateral carpal tunnel syndrome) or autonomic dysfunction in patients with ventricular hypertrophy and/or heart failure with preserved ejection fraction, given that 31% of our patients presented this association, which is a finding that has been previously described in the literature.^13^

The biomarkers BNP and troponin are important for demonstrating and quantifying myocardial aggression in patients with amyloidosis. Moreover, these laboratory tests are also used for prognostic assessment of these patients, and they have been observed in part of our patients, denoting both the severity of the disease and the character of its continuous active aggression. Our data corroborate the literature, which identifies BNP, NT-pro-BNP, and troponin as potential diagnostic and prognostic tests in suspected or confirmed cases of amyloidosis. When associated with symptoms of heart failure or polyneuropathy, altered troponin should be considered a warning sign for systemic amyloidosis in the absence of an acute ischemic or inflammatory condition or of other diseases such as atrial fibrillation and chronic kidney disease.^10,12^

ECG alterations usually occur late, once there is already severe cardiac impairment, but, when present, they assist in diagnosis. ECG suggests diagnosis when signs of left atrial overload, first-degree atrioventricular block, pseudo-inactive anteroseptal area pattern, diminished septal strength, and low voltage are concomitantly observed, especially when this is discordant with the myocardial hypertrophy found on the echocardiogram or cardiac resonance.^17^

ECG and 24-hour Holter showed evidence of atrioventricular and intraventricular conduction disorders in nearly half of patients (Tables 2 and 3), followed by low voltage and inactive area. In our study, we observed a higher incidence of ECG alterations than in another Brazilian study, where 56% of patients presented these findings, probably due to the fact that there were more patients with a mutation whose phenotypic presentation is predominantly neurological (Val50Met, early form). Nevertheless, in both studies, the incidence of conduction disorders was similar in affected patients.^18^

As it is a deposition, infiltrative, and also inflammatory disease, conduction disorders are commonly found. The data in the literature suggest that atrioventricular blocks are among the most frequent alterations, occurring in 38% of patients.^20^ Nevertheless, in our study, we did not identify this prevalence, possibly due to the fact that we included patients with earlier forms, by actively screening asymptomatic carriers of mutations, family members of patients with confirmed diagnosis, and due to different mutations included in different studies. In comparison with another Brazilian study, by Queiroz et al., with 51 patients, their data were more similar to the data we found, with approximately 13.7% of patients with atrioventricular blocks and 19.6% with intraventricular blocks.^20^

The prevalence of atrial fibrillation grows exponentially with age; there are epidemiological studies indicating that it is 3.7% to 4.2% in those between the ages of 60 and 70 years and around 7% to 8% at 75 years of age.^20,21^ It was, therefore, expected that, in our sample, the incidence of fibrillation in patients with wild-type cardiac amyloidosis would be higher than in the hereditary form, given the difference in age at the time of disease diagnosis in both groups. Indeed, prevalence was much higher than the global population data, as 7% of patients with the ATTRh form and 38.5% of patients with the ATTRw form had this arrhythmia. Even so, our prevalence was lower than that found in the sample of Donnellan et al.,^22^ who observed atrial fibrillation in 69% of patients with transthyretin cardiac amyloidosis. These findings suggest that fibrillation can be a marker of the disease, especially in patients with no apparent cause that would explain this arrhythmia.

In the same manner as atrial fibrillation, frequent supraventricular ectopic beats (defined as > 30 ectopics per hour) and atrial tachycardias are more prevalent with increasing age; this was also shown in our sample, where 61.5% of patients with the ATTRw form had one or more of these forms of arrhythmias, alone or concomitantly.

While they are less observed than atrial arrhythmias in this population, ventricular arrhythmias are also found, with a spectrum ranging from rare ectopic beats (defined as < 10 ectopics per hour) to episodes of ventricular tachycardia, which are nonsustained. In one study, complex ventricular arrhythmia was observed in 17% of patients with ATTR amyloidosis and in 27% of those with AL amyloidosis. In our study, we also observed a higher percentage incidence of ventricular arrhythmia in the AL form (13.6%) than in the ATTR form (9.6%), but the difference was not statistically significant, and both had a lower prevalence than in the aforementioned study.^23^ Accordingly, amyloidosis should also be considered as a differential diagnosis in patients who have cardiac arrhythmias without an apparent cause.

Imaging exams contribute to the recognition of cardiac amyloid infiltration, as they assess the presence and severity of ventricular hypertrophy, as well as systolic and diastolic dysfunction.^24-26^ Nonetheless, the most typical morphological and functional alterations are observed at a more advanced stage of the disease, and they are correlated with the amount of systemic amyloid deposition and worsening of clinical signs and symptoms.^27,28^

Regarding the echocardiography findings in our sample, we observed that patients with ATTRw had a higher degree of diastolic dysfunction than those with ATTRh, which can be explained by the longer disease duration and the higher prevalence of symptoms.

When comparing forms of cardiac amyloidosis, it has been reported that patients with the ATTRw form are characterized by greater left ventricular hypertrophy, lower ejection fraction, and lower longitudinal strain in the ATTRw and AL forms than in the ATTRh form.^2^ In our sample, we did not find significant differences between types in terms of hypertrophy or function. Patients with ATTRw with longer disease duration had larger left ventricular and left atrial diastolic diameters (p = 0.012 and 0.001 respectively).

More advanced echocardiography techniques, such as strain and strain rate derived from speckle tracking, can assist in assessment of torsional motion of the heart and facilitate in differentiation between cardiac amyloidosis and hypertrophic cardiomyopathy, but they are not routinely applied in clinical practice.^24^ This finding suggests that there is a need for diagnostic complementation with other imaging methods, such as cardiac magnetic resonance and bone scintigraphy.

Cardiac magnetic resonance is another method of imaging evaluation that provides information about cardiac function and morphology in patients with amyloidosis.^25,26^ The most specific delayed enhancement pattern is diffuse, circumferential left ventricular subendocardial enhancement.^29,30^ In our study, we obtained information regarding the presence of delayed enhancement; however, due to the lack of standardization, it was not possible to conduct segmental evaluation. Nonetheless, of the 55 patients with this information, 71% had late enhancement in a non-ischemic pattern.

The asymmetric pattern of myocardial hypertrophy in patients with ATTR amyloidosis differs from that in patients with the AL form, which is generally symmetrical. In a study of 263 patients with ATTR amyloidosis confirmed by scintigraphy with PYP-^99m^Tc and compared with 50 patients with the AL form, the presence of asymmetric hypertrophy was observed in 79% of cases in the ATTR form; it was symmetric in 18% and absent in 3%. The delayed enhancement pattern was 29% subendocardial and 71% transmural.^27^ In our sample, we observed a lower incidence of asymmetric hypertrophy in the ATTR form, as well as a greater incidence than expected in the AL form.

In 2016, Gillmore et al. demonstrated that myocardial scintigraphy with PYP-^99m^Tc allows for reliable diagnosis of ATTR amyloidosis without the need for histological confirmation, or be it, without requiring cardiac biopsy, in patients without monoclonal peaks.^31^ In our sample, data on cardiac scintigraphy were limited due to the heterogeneity of the protocol inherent in the implementation of the method. Even so, it was possible for us to observe that, as in Gilmore’s work, the AL form presented uptake at 3 hours that was significantly lower than the ATTR form. This is currently the validated method with best sensitivity and specificity for transthyretin amyloidosis, once monoclonal gammopathies have been ruled out.^32^

As previously described by other authors, we observed cardiac amyloidosis as a great mimicker of other forms of heart disease, manifesting in various manners, including the following: heart failure, with both reduced (more advanced conditions) and preserved ejection fraction; hypertrophic cardiomyopathy (both symmetric and asymmetric); atrial and ventricular arrhythmias; and conduction system disorders, thus simulating other diseases and making it difficult to diagnose.^11,12^

It was possible to observe important data, such as the finding that the most prevalent mutation associated with cardiac involvement in Brazil was Val142Ile, in spite of what might have been expected given that Brazil was colonized by Portugal, where Val50Met predominates, as well as the longer time to diagnosis of the ATTRw form in Brazil. This study also underscores the importance of recognizing warning signs to detect cardiac involvement in amyloidosis, such as biomarker levels; the presence of polyneuropathy in patients with heart failure with preserved ejection fraction and/or hypertrophic cardiomyopathy; the range of possible ECG changes; and the high prevalence of arrhythmias in this population (especially atrial fibrillation, with all its implications). Moreover, we observed the importance of imaging methods as markers of cardiac involvement by means of left ventricular global longitudinal strain, uptake after 3 hours in scintigraphy with pyrophosphate, and presence of late enhancement in cardiac magnetic resonance.

### Limitations

This was a retrospective single-center study, with a convenience sample, conducted in a cardiology referral center. Some of the patients were forwarded from other services, which did not always have the same standards for performing some exams.

## Conclusions

Amyloidosis is a disease with a heterogeneous phenotypic presentation. Early diagnosis requires a high degree of clinical suspicion, and there is a long interval between onset of symptoms and diagnosis. Biomarkers, ECG, and imaging methods are fundamental to investigation, especially when they are associated with suggestive clinical history, such as polyneuropathy concomitant to heart failure with preserved ejection fraction, and specific genetic investigation.
